# Current perspectives on proton and electron transfer pathways in photosystem II water oxidation

**DOI:** 10.1093/pcp/pcaf107

**Published:** 2025-08-30

**Authors:** Hiroshi Ishikita, Keisuke Saito

**Affiliations:** Department of Applied Chemistry, The University of Tokyo, 7-3-1 Hongo, Bunkyo-ku, Tokyo 113-8654, Japan; Research Center for Advanced Science and Technology, The University of Tokyo, 4-6-1 Komaba, Meguro-ku, Tokyo 153-8904, Japan; Department of Applied Chemistry, The University of Tokyo, 7-3-1 Hongo, Bunkyo-ku, Tokyo 113-8654, Japan; Research Center for Advanced Science and Technology, The University of Tokyo, 4-6-1 Komaba, Meguro-ku, Tokyo 153-8904, Japan

**Keywords:** proton-coupled electron transfer, redox potential, electronic coupling, low-barrier hydrogen bond, oxygen evolution, protein crystal structure

## Abstract

Photosystem II catalyzes the light-driven oxidation of water, progressing via sequential oxidation states (S-states) of the Mn_4_CaO_5_ cluster. Among structural snapshots of intermediate S-states obtained using X-ray free-electron laser (XFEL) crystallography, two-flash XFEL structures assigned to the S_3_ state reveal an additional oxygen atom (O6) near the O5 site of the cluster, leading to proposals that O6 is incorporated as a new substrate water molecule during the S_2_ to S_3_ transition. However, recent re-analyses of the XFEL data highlight potential complications, including conformational heterogeneity, refinement bias, and possible radiation-induced artifacts. In addition, many proposals have been put forwarded without evaluating associated proton and electron transfer processes, despite the fact that water oxidation involves the stepwise removal of protons and electrons. Here, we shed light on electron and proton transfer events during the photocycle by summarizing mechanistic proposals, including those in which O6 is not incorporated. If the remaining reduced site, Mn1(III), is oxidized during the S_2_ to S_3_ transition, this step encounters difficulties due to its high redox potential and poor electronic coupling with the electron acceptor, D1-Tyr161 (TyrZ). Efficient proton transfer requires pre-existing H-bond networks, which are absent near O5 and O6, imposing kinetic penalties on proton release. Assigning O6 as a substrate oxygen would imply that O5 is the other substrate, requiring its deprotonation earlier in the Kok cycle.

## Introduction

**Figure 1:**
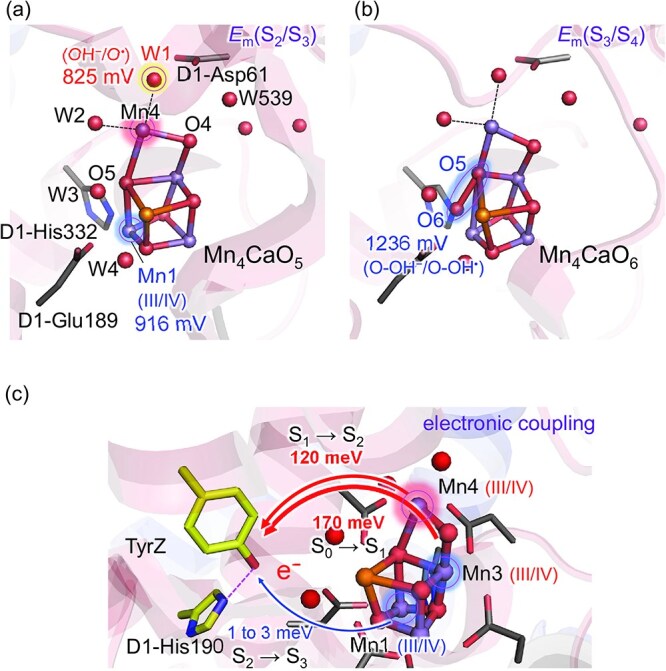
Redox potential and electronic coupling for the Mn_4_CaO_5_ cluster. (a) Structure of the Mn_4_CaO_5_ cluster. Highlighted sites with numerical values indicate redox potential values in the S_2_ to S_3_ transition. (b) Structure of the Mn_4_CaO_6_ cluster. The highlighted site with a numerical value indicates the redox potential value in the S_3_ to S_4_ transition. (c) Electronic coupling between the OEC and TyrZ: 170 meV in the S_0_ to S_1_ transition, 120 meV in the S_1_ to S_2_ transition (both via Mn4), and 1–3 meV in the S_2_ to S_3_ transition (via Mn1).

At the heart of photosystem II (PSII) lies the oxygen-evolving complex (OEC), a catalytic center composed of the Mn_4_CaO_5_ (Umena et al. 2011) [or Mn_4_CaO_6_ when incorporating an additional oxygen atom (Suga et al. 2017)] cluster, which facilitates the sequential four-electron oxidation of two substrate water molecules (Fig. 1). This reaction proceeds through the Kok cycle (Kok et al. 1970), advancing stepwise through five intermediate oxidation states (S-states) (S_0_, S_1_, S_2_, S_3_, and S_4_). Each S-state transition involves one-electron oxidation of the OEC, except for the S_4_ to S_0_ transition, during which molecular oxygen is released. Upon oxidation of the Mn_4_CaO_5_ cluster, electron transfer occurs to oxidized redox-active D1-Tyr161 (TyrZ-O^•^), which is generated via electron transfer to the oxidized chlorophyll pair [P_D1_P_D2_]^•+^ in the D1 and D2 proteins. Proton release toward the lumenal protein surface accompanies the S-state transitions in the following stoichiometry: 1 for S_0_ to S_1_, 0 for S_1_ to S_2_, 1 for S_2_ to S_3_, and 2 for S_3_ to S_0_ (Rutherford and Boussac 2004, Dau and Haumann 2008, Shen 2015, Ifuku and Noguchi 2016, Kaur et al. 2019, Yano et al. 2024).

A major milestone in elucidating water oxidation was the determination of the 1.9 Å resolution crystal structure, which revealed the Mn_4_CaO_5_ cluster composed of four Mn ions (Mn1–Mn4), one Ca^2+^, four ligand water molecules (W1–W4), and five bridging oxo ligands (O1–O5) (Kawakami et al. 2011, Umena et al. 2011). This configuration was later confirmed to represent primarily the S_1_ state, based on structures obtained using X-ray free-electron laser (XFEL) (Suga et al. 2015) or significantly lower X-ray doses (Tanaka et al. 2017).

Subsequent XFEL studies successfully captured light-induced structural snapshots corresponding to higher S-states, including S_2_ and S_3_, using one-flash (1F-XFEL) and two-flash (2F-XFEL) illumination, respectively (e.g. Suga et al. 2017, Kern et al. 2018, Suga et al. 2019, Hussein et al. 2021, Bhowmick et al. 2023, Li et al. 2024). A striking feature of the 2F-XFEL structures, assigned to the S_3_ state, is the appearance of an additional oxygen atom, designated O6 (Suga et al. 2017) or Ox (Kern et al. 2018), located near O5 at the quasi-center of Mn1 and Mn4. In these structures, O5 and O6 exhibit an unusually short distance [e.g. 1.5 Å (Suga et al. 2017), 1.9 Å (Suga et al. 2019), and 2.1 Å (Kern et al. 2018)], suggesting that O6 might participate in O–O bond formation.

Several theoretical models have been proposed to rationalize how O6 might be incorporated during the S_2_ to S_3_ transition. These models include: external water insertion at Mn1, as proposed by Siegbahn, where an incoming water molecule binds to Mn1, is deprotonated, and becomes O6 (Siegbahn 2013); internal ligand rearrangement, where W3 migrates from Ca^2+^ to Mn4 to form O6 (e.g. Bovi et al. 2013, Shoji et al. 2015, Ugur et al. 2016, Allgöwer et al. 2022); concerted pivot-like rotations of W1 and W2 around Mn4, indirectly leading to O6 formation (e.g. Askerka et al. 2015, Retegan et al. 2016).

While these models propose seemingly elegant pathways for O6 incorporation, they rarely address the coupled energetics of electron transfer from the Mn_4_CaO_5_ cluster to TyrZ-O^•^, nor the accompanying proton release (1H^+^) toward the bulk solvent. Since under the ‘high oxidation state paradigm’ [in contrast to the ‘low oxidation state paradigm’ (Gatt et al. 2012)], the S_2_ state is typically formulated as Mn(IV)_3_Mn(III), many models assume oxidation of the remaining Mn1(III) site during the S_2_ to S_3_ transition. However, Mn1 possesses the highest redox potential (*E*_m_), due to the absence of a functional pre-existing H-bond network near O6 that could support efficient proton-coupled electron transfer (Mandal et al. 2020a). This lack of a well-defined H-bond pathway is evident from both high-resolution and 1F-XFEL structures.

The lack of such pre-organized H-bond networks near both O5 and O6 imposes a substantial kinetic barrier to deprotonation, requiring transient formation of an H-bond network, a thermally activated and entropically disfavored process (Stuchebrukhov 2009). Somewhat surprisingly, none of the theoretical models proposing O6 incorporation have explicitly provided a potential energy profile for the relevant H-bond between O6 and a putative proton acceptor. Such a profile is essential to demonstrate whether the proton transfer can occur via a low-barrier H-bond (LBHB), a standard criterion for validating proton transfer reactions in proteins (Schutz and Warshel 2004).

In nearly all well-characterized proton transfer events in proteins, the existence of LBHBs has been readily demonstrated directly from the original protein structures, without requiring artificial structural manipulation. Examples includes proton transfer between the retinal Schiff base and its counterion in microbial rhodposins (Tsujimura and Ishikita 2021, Tsujimura et al. 2022); between *p*-coumaric acid and Glu46 in photoactive yellow protein (Saito and Ishikita 2013, Tsujimura et al. 2022); and between quinone (Q_B_) and His-L190 in photosynthetic reaction centers from purple bacteria (Sugo et al. 2021). Even within PSII, LBHBs have been reproducibly demonstrated between O4 and the O4-water chain during the S_0_ to S_1_ transition (Saito et al. 2015); between W1 and D1-Asp61 during the S_2_ to S_3_ transition (Kawashima et al. 2018b, Saito et al. 2020, Saito et al. 2023); between TyrZ and D1-His190 (Saito et al. 2011, Kawashima et al. 2018a); and between Q_B_ and D1-His215 (Saito et al. 2013).

Against this background, the continued lack of the potential-energy profile for an H-bond involving O6, even as late as 2025, stands out as highly unusual. It raises the question of whether LBHB formation is fundamentally absent for O6, or whether unfavorable results have been selectively omitted from publication.

The hypothesis that O6 is incorporated during the S_2_ to S_3_ transition has become a widely accepted paradigm in the field, and numerous studies have been conducted under this assumption. However, this dominant view has led to a narrowing of discussion, where alternative mechanisms that do not involve O6 incorporation, even though scientifically plausible, remain underrepresented in the literature. To our knowledge, no review has systematically organized and presented such alternative perspectives.

Surprisingly, among the many theoretical studies aimed at elucidating the water-splitting mechanism, those that address proton transfer remain limited. Evaluating the potential-energy profile along an H-bond proposed as a proton transfer pathway is a standard and essential step to evaluate whether such transfer is energetically possible (Perrin and Nielson 1997, Schutz and Warshel 2004). However, for PSII, only a small number of studies have faithfully carried out this type of analysis (e.g. Saito et al. 2011; Saito et al. 2015). Furthermore, aside from work by a very few specific groups, no theoretical studies have simultaneously investigated the essential components of electron transfer, namely, redox potentials (e.g. Mandal et al. 2020b) and electronic coupling (e.g. Tamura et al. 2023, Tamura et al. 2025).

This striking absence, in turn, suggests that many mechanistic proposals are driven primarily by structural considerations—selecting oxygen atoms in structurally eye-catching positions (e.g. O5) and constructing hypotheses around them—rather than beginning with an evaluation of the thermodynamics and kinetics of proton and electron removal. From a physical chemistry perspective, this situation for PSII, i.e. the neglect of proton and electron transfer, is unusual, given that the photocycle proceeds via the stepwise abstraction of four protons and four electrons. It should be noted that water molecules are incorporated only after changes in the electrostatic environment, triggered by these charge-removal events, generate a driving force, *not the other way around*. Thus, the lack of studies focused on these aspects indicates a notable ‘imbalance’ in the field, despite their central importance to the underlying chemistry of water oxidation. In this context, it is essential to revisit the commonly proposed mechanisms from the often-overlooked standpoint of proton and electron transfer.

Here, we provide a complementary viewpoint by summarizing mechanistic proposals, including those in which O6 is not incorporated, focusing on proton and electron transfer pathways during the S_2_ to S_3_ transition.

## Challenges in Interpreting O6 Incorporation from XFEL Structures

Since Suga et al. reported a 2F-XFEL structure in 2017, in which a new μ-oxo bridge, designated O6, was modeled between Mn1 and Mn4 (Suga et al. 2017), 2F-XFEL structures, widely interpreted as representing the S_3_ state, have played a central role in motivating models for O6 incorporation during the S_2_ to S_3_ transition. However, the assignment of O6 was not based on well-defined positive electron density in the 2m*F*_o_ − D*F*_c_ map. Instead, it was modeled based on a positive peak in the m*F*_o_ − D*F*_c_ map near O5, unlike earlier high-resolution crystallographic studies (Umena et al. 2011), in which all components of the Mn_4_CaO_5_ cluster were explicitly resolved in 2*F*_o_ − *F*_c_ maps.

Several recent studies have raised important concerns regarding this interpretation and suggest that the structural data may be more ambiguous than initially appreciated. One alternative proposal by Petrie et al., which is based on the low Mn oxidation state paradigm, suggests that the observed electron density assigned to O6 in the 2F-XFEL structure may represent a superposition of two distinct conformations (Petrie et al. 2020). In their model, the S_3_ state retains a low oxidation configuration (Mn(III)_2_Mn(IV)_2_ rather than Mn(IV)_4_), and the O6 site corresponds not to a bridging μ-oxo ligand but to a water-derived ligand bound asymmetrically to either Mn1 or Mn4. This interpretation implies that the electron density near the O6 region in the XFEL structure does not reflect to a single, well-defined intermediate, but rather an averaged signal from a heterogeneous mixture of species. This view challenges the uniform mechanistic interpretation of the O6 incorporation and underscores the difficulty of assigning substrate positions solely based on such structural data.

This structural heterogeneity is further emphasized by the fact that 2F-XFEL crystals often contain substantial fractions of multiple S-states simultaneously [e.g. ~ 51% of S_3_ and ~49% of S_2_ (Suga et al. 2019)], which adds to the complexity of interpreting the short O. . .O distances identified in the O5 and O6 region.

Wang et al. reanalyzed the original XFEL data and demonstrated that the electron density features assigned to O6 are not supported by independent difference Fourier maps (Wang et al. 2021). They argued that the observed data can be fully explained using a five-oxygen ligand model (Mn_4_CaO_5_) without requiring an additional O6 atom, suggesting that O6 assignment may have resulted from model bias introduced during crystallographic refinement (Wang et al. 2021). Notably, in their more recent analysis, they also reexamined high-resolution structures, including the 2.0 Å XFEL structure by Suga et al. (2019) and the 1.7 Å cryo-EM structure by Hussein et al. (2024), and showed that neither structure unambiguously supports the presence of O6 (Wang 2025).

Beyond these issues, radiation damage remains a further complicating factor in XFEL data interpretation. Molecular modeling studies by Amin et al. have suggested that even during the ultra-short pulse durations used in XFEL (10–50 fs), local radiation-induced ionization events may induce significant atomic displacements, particularly around heavy metal centers such as the Mn_4_CaO_5_ cluster (Amin et al. 2016). Such processes may distort bond lengths and give rise to short O. . .O distances not representative of physiological intermediates. Thus, some of the structural features observed under XFEL conditions may partly reflect radiation effects rather than intrinsic intermediates of the catalytic cycle.

A separate, yet related structural feature emerges from the exceptionally short O4. . .W539 [W539 in Umena et al. (2011) and W567 in Suga et al. (2017)] distance [~2.32 Å (Suga et al. 2017)] observed in 2F-XFEL structures. Suga et al. emphasized that ‘The average distance between W567 and O4 upon 2F illumination in the two monomers was determined to be 2.32 Å. This distance is extremely short, which may suggest that W567 is a hydroxide ion (OH^–^) rather than a water (H_2_O) in the S_3_ state’ (Suga et al. 2017). This inspired Corry and O’Malley to propose that, based on calculations in the absence of the PSII protein environment, O4 was OH^−^ in S_2_ (Corry and O’Malley 2020). However, as pointed out in Saito et al. (2022), in the presence of the PSII protein environment, OH^−^ based at O4 is unstable and does not exist in S_2_ (Saito et al. 2015).

For the short O. . .O distance (2.32 Å) in the 2F-XFEL structure (Suga et al. 2017), quantum mechanical/molecular mechanical (QM/MM) calculations by Mandal et al. reproduced the distance only when the H-bond partner water molecule, W539 (W567) is a hydroxyl radical (OH^•^), as such a distance is too short even for a LBHB, raising the possibility that this observation may partly reflect radiation-induced processes rather than fully equilibrated catalytic intermediates (Mandal et al. 2021).

Taken together, these recent analyses collectively caution against overinterpreting the 2F-XFEL structures. Instead, the XFEL structures may represent a mixture of conformational substates, potentially influenced by sample heterogeneity, radiation effects, or crystallographic model bias. Until the mechanistic identity of the proton donor and oxidation sites during the S_2_ to S_3_ transition is fully resolved, the role—or even the existence—of O6 incorporation remains an open question that warrants further careful evaluation, rather than being prematurely fixed as an established mechanistic step.

## Electron Transfer

For electron transfer involving the Mn_4_CaO_5_ cluster in PSII, the key principles follow Marcus theory, where three major factors govern electron transfer: (i) the Gibbs free energy change (*Δ*G), (ii) electronic coupling, and (iii) solvent (outer-sphere) reorganization energy.

First, identification of the physiologically relevant oxidation site requires careful evaluation of *Δ*G, which corresponds to the difference in *E*_m_, or, equivalently, the difference in the highest occupied molecular orbital (HOMO) energy between the electron donor and acceptor. Since the Mn site with the lower *E*_m_ is preferentially oxidized, evaluation of *E*_m_ values for each candidate site is essential for identifying which Mn ion is oxidized.

Second, unlike isolated catalytic metal clusters where diffusible oxidants may approach from any direction, the Mn_4_CaO_5_ cluster in PSII is embedded in a fixed protein matrix and oxidized exclusively by its intrinsic electron acceptor, TyrZ. Therefore, evaluation of electronic coupling between the electron donor, Mn_4_CaO_5_, and the electron acceptor, TyrZ, is essential to establish whether a functional electron transfer pathway exists. This electronic coupling differs substantially among candidate Mn sites due to variations in their electronic structures and exchange interactions (e.g. Mn1(III)Mn2(IV)Mn3(III)Mn4(III) in S_0_). If the calculated coupling strength is significantly weaker than would be expected from experimentally observed electron transfer rates, the proposed oxidation site should be reconsidered.

In contrast, solvent (outer-sphere) reorganization energy is expected to vary less significantly among the individual oxidation sites because it primarily reflects the overall dielectric response of the protein–solvent environment to the net charge change of the Mn_4_CaO_5_ cluster as a whole, rather than the specific identity of the oxidized Mn center.

With these considerations established, we next evaluate how these principles apply to the proposed O6 incorporation mechanism during the S_2_ to S_3_ transition.

### Relevant oxidation state in the 2F-XFEL structures

The incorporation of an additional oxygen atom, O6, into the Mn_4_CaO_5_ cluster during the S_2_ to S_3_ transition has been proposed based on 2F-XFEL structures, which are assigned to represent the S_3_ state. These structures revealed short distances between O5 and O6: 1.9 Å in the Suga et al. 2F-XFEL structure (Suga et al. 2019) and 2.1 Å in the Kern et al. 2F-XFEL structure (Kern et al. 2018). These distances are significantly shorter than typical H-bonds (∼2.8 Å), even shorter than LBHBs (~2.4 to 2.5 Å), yet longer than covalent O–O single bonds (~1.4 to 1.5 Å), suggesting a highly unusual bonding configuration at this stage.

QM/MM calculations by Mandal et al. have directly addressed the nature of the O5. . .O6 interaction. The observed short O5. . .O6 distances can only be reproduced if O6 is assigned as a deprotonated hydroxyl radical (O^•–^), rather than as neutral water (H_2_O) or hydroxide (OH^−^) (Mandal et al. 2020b). Due to the presence of O^•–^, one of the Mn sites cannot be oxidized, (Mn1, Mn2, Mn3, Mn4) = (IV, IV, IV, III) in S_3_, indicating that Mn4 remains in the Mn(III) state during the S_2_ to S_3_ transition. In this scenario, ligand-centered oxidation (e.g. Messinger et al. 2001), rather than Mn-centered oxidation, accounts for the additional oxidation equivalent in this step. In contrast, mechanistic models that assume O6 incorporation as H₂O or OH^−^ can maintain Mn-centered oxidation, leading to a Mn(IV)_4_ configuration in S_3_. However, as a trade-off, these models fail to reproduce the observed short O5. . .O6 distances in the XFEL structures.

Taken together, if one respects the structural data quantitatively, the 2F-XFEL structures point to an S_3_ state of Mn(IV)_3_Mn(III) with O6 already oxidized to O^•–^, supporting a ligand-centered oxidation mechanism. If, however, one chooses to disregard these details, specifically, the refined O5. . .O6 distance, and treats the structures only qualitatively, then the canonical Mn-centered oxidation sequence leading to Mn(IV)_4_ may still appear plausible.

However, one of the central achievements originally emphasized by Suga et al. in the 2019 structure was the refinement of the O5. . .O6 distance from 1.5 Å in their earlier 2017 structure (Suga et al. 2017) to 1.9 Å (Suga et al. 2019). Given the quantitative refinement emphasized by Suga et al. (2019), the structural data more strongly favor the ligand-centered oxidation scenario. In this context, it is especially important for theoretical researchers to approach the original structural data, including coordinates and B-factors, with the highest care and integrity, so as to avoid the temptation of adjusting structures to fit preexisting mechanistic models.

It should be noted that the QM/MM study by Mandal et al. does not suggest that S_3_ is Mn(IV)_3_Mn(III) with O6 already oxidized to O^•–^ (Mandal et al. 2020b). Rather, it suggests that if the refined O5. . .O6 distance from the 2F-XFEL structure (= 1.9 Å) (Suga et al. 2019) is taken as quantitatively accurate and interpreted as representing an intact S₃ state, then O6 would necessarily be in the O^•–^ form.

### Electronic coupling required for oxidation of Mn_4_CaO_5_ by TyrZ

In contrast to isolated catalytic metal clusters, which can be oxidized by diffusible oxidants from any direction, the Mn_4_CaO_5_ cluster in PSII is oxidized exclusively by its intrinsic electron acceptor, TyrZ. Both are embedded in fixed orientations in the protein matrix, which constrains the electron transfer pathway. Although evaluating the electronic coupling between Mn_4_CaO_5_ and TyrZ is essential for understanding the sequential S-state transitions, its quantitative analysis remains technically challenging, and only a limited number of studies have addressed it directly (e.g. Tamura et al. 2023, Tamura et al. 2025).

In the S_0_ to S_1_ transition, although the net change in Mn valence suggests Mn3(III) is oxidized, the initial electron transfer is from Mn4(III) to TyrZ, followed by intracluster electron redistribution from Mn3 to Mn4. This two-step mechanism is supported by the large electronic coupling between Mn4 and TyrZ [~170 meV (Tamura et al. 2023)] (Fig. 1c). Similarly, in the S_1_ to S_2_ transition, the ET again proceeds from Mn4(III), and the coupling remains large [~120 meV (Tamura et al. 2023)] (Fig. 1c). These couplings are consistent with observed fast (~30 to 45 μs) and slightly slower (~70 μs) ET timescales for the S_0_ to S_1_ and S_1_ to S_2_ transitions, respectively (Dau and Haumann 2008, Shimizu et al. 2018).

However, during the S_2_ to S_3_ transition, if Mn1(III) is assumed to be the oxidation site, as required by several O6 incorporation models, the electronic coupling between Mn1 and TyrZ becomes dramatically weaker (~1 to 3 meV) (Fig. 1c), more than 100-fold smaller than in the previous transitions (Tamura et al. 2025). This severe reduction in coupling arises because the HOMO is not localized on Mn1 but is delocalized over Mn2 and Mn4, due to strong antiferromagnetic exchange interactions. Moreover, unlike Mn4, which is connected to TyrZ via D1-Asp170, forming a key superexchange pathway (Tamura et al. 2023), Mn1 lacks a similarly effective bridge. Although D1-Glu189 coordinates Mn1, its orbital contribution to the TyrZ pathway is minimal, further weakening the coupling.

This weak Mn1-to-TyrZ coupling is difficult to reconcile with the observed ET timescale for the S_2_ to S_3_ transition (~350 μs [Sakamoto et al. 2017]), which is only moderately slower than those of the S_0_ to S_1_ and S_1_ to S_2_ transitions. The discrepancy suggests that Mn1 oxidation, as postulated in many O6 insertion models, is kinetically disfavored when considering electronic coupling.

Thus, models that invoke Mn1(III) oxidation as part of the S_2_ to S_3_ transition with O6 incorporation face a fundamental challenge: despite potentially favorable shifts in *E*_m_ for Mn1(III/IV) due to nearby OH^−^ binding at O6, the electron tunneling pathway itself is bottlenecked by extremely weak coupling. Nevertheless, many theoretical studies have carelessly adopted Mn1(IV) in S_3_ without evaluating the electronic coupling required for Mn1(III) oxidation. It should be emphasized, especially for experimental researchers, that obtaining a QM/MM optimized structure does not, by itself, validate the assumed oxidation state assignment. In quantum chemical calculations, various oxidation states, such as Mn1(IV), can readily be generated simply by adjusting the total charge and spin multiplicity at the outset, provided the initial configuration is not grossly unreasonable. However, convergence to a stable structure does not necessarily indicate that the resulting state is chemically or mechanistically relevant. Therefore, independent evaluation of electronic coupling and *E*_m_ for each oxidation site is essential to assess the plausibility of the modeled oxidation state.

As a result, a serious conflict arises between such assumptions in many theoretical studies, considering S_3_ as Mn1(IV) with OH^−^ at O6, and the experimentally observed electron transfer kinetics governing the S_2_ to S_3_ transition.

### Redox potential in proton-coupled electron transfer, including the S_2_ to S_3_ transition

Redox potential provides crucial energetic insights into which sites are energetically most favorable for oxidation during each step of the S-state transitions. In the S_2_ to S_3_ transition, proton release has been identified as the rate-limiting step (Dau and Haumann 2008). Therefore, proton-coupled electron transfer plays a central role in determining the redox energetics of this step. The oxidation of the W1 water ligand, which is coordinated to Mn4(III) and forms a LBHB with D1-Asp61 (Kawashima et al. 2018b, Saito et al. 2020, Saito et al. 2023), is both structurally and energetically highly favorable in the S_2_ to S_3_ transition. Deprotonation of W1 to D1-Asp61 facilitates proton-coupled electron transfer by efficiently removing the accumulating positive charge as H^+^, thereby significantly lowering *E*_m_ required for W1 oxidation (Mandal et al. 2020a). This principle is further supported by electrochemical studies of synthetic Mn oxides, where carboxylate-mediated oxidation exhibits similarly lowered *E*_m_ (Hayashi et al. 2016). Because the H-bond network extending from W1 allows proton release to compensate for charge accumulation, the *E*_m_ for W1 oxidation remains relatively low (~830 mV) (Fig. 1a). In contrast, Mn1(III) oxidation requires a substantially higher *E*_m_ (~920 mV) due to the absence of a comparable proton-conducting H-bond network (Mandal et al. 2020a).

Notably, if oxidation of Mn1(III) were to occur, forming Mn(IV)_4_ in S_3_, then subsequent oxidation of the O5. . .O6 moiety would subsequently be required to enable O–O bond formation, making it the redox-active site during the S_3_ to S_4_ transition. However, for the S_3_ conformation with Mn(IV)_4_ and OH^−^ assigned at O6, the calculated *E*_m_(S_3_/S_4_) is unusually high (~1240 mV), suggesting that electron abstraction from TyrZ-O^•^ is unlikely to occur to initiate O–O bond formation (Mandal et al. 2022).

Taken together, oxidation of Mn1(III) is disfavored both *kinetically*, due to the very weak electronic coupling to TyrZ (1–3 meV) (Tamura et al. 2025), and *energetically*, due to its significantly higher *E*_m_ (Mandal et al. 2020a). Of the three major factors influencing electron transfer in Marcus theory, two (*Δ*G and electronic coupling) are clearly inconsistent with Mn1 oxidation in the S_2_ to S_3_ transition. (As noted above, solvent reorganization energy is expected to vary only modestly among individual Mn sites.) These findings strongly challenge the involvement of Mn1 as the redox-active site during this step.

In contrast, the chemical principle—interplay between electron transfer and proton transfer via a well-defined H-bond network lowers the *E*_m_, is consistent with other proton-coupled electron transfer steps, in the S_0_ to S_1_ transition, where Mn3 oxidation is coupled to O4 deprotonation via a LBHB, and in the S_2_ to S_3_ transition, where W1 deprotonation to D1-Asp61 similarly lowers *E*_m_ (Mandal et al. 2020a). In both cases, the redox-active site is directly linked to a well-defined proton-conducting H-bond network, as consistently observed in high-resolution PSII structures (Umena et al. 2011, Suga et al. 2015). Notably, the kinetic isotope effect is even more pronounced in the S_2_ to S_3_ transition than in the S_0_ to S_1_ transition (Dau and Haumann 2008, Shimizu et al. 2018), further supporting that proton transfer occurs preferentially along the pre-existing W1. . .D1-Asp61 H-bond network, rather than transiently formed H-bonds hypothesized near the O5. . .O6 moiety.

Given that the kinetic, energetic, and structural data do not predominantly support proton-coupled electron transfer mechanisms invoking Mn1(III) oxidation coupled to O6 deprotonation over the other two well-established proton transfer pathways (O4 deprotonation and W1 deprotonation to D1-Asp61, see below), current research outcomes still make it difficult to rationalize such models for the S_2_ to S_3_ transition, even though the proposed O6 incorporation may appear mechanistically attractive in principle.

## Proton Transfer

In contrast to electron transfer, proton transfer involves a particle 1836 times more massive than an electron. This fundamental mass difference means that, unlike electrons, protons cannot delocalize or tunnel over long distances unless guided by a pre-formed and continuous H-bond network. Therefore, the structural continuity of the H-bond network is a prerequisite for kinetically feasible proton transfer.

Nevertheless, many theoretical studies on PSII (e.g. Siegbahn 2013, Petrie et al. 2015) evaluate only the energy difference between protonated and deprotonated states of the Mn_4_CaO_5_ cluster, essentially corresponding to the intrinsic p*K*_a_ of the proton donor site alone. However, unless the donor site is directly exposed to bulk solvent, this approach does not even adequately capture the energetics of deprotonation, in particular, in the protein environment.

In the case of the Mn_4_CaO_5_ cluster, which is embedded in the protein environment and shielded from bulk solvent, proton release requires not only removal of the proton from the donor site but also its transfer onto an acceptor site. Thus, the energetically relevant quantity is the energy difference between the pre-proton transfer state (proton localized on the donor) and the post-proton transfer state (proton localized on the acceptor). This energy difference reflects the p*K*_a_ difference between donor and acceptor sites—not simply the p*K*_a_ of the donor alone. This scenario is analogous to the evaluation of electron transfer energetics or charge separation energetics (Ishikita and Saito 2025). In the former case, electron transfer energetics, the evaluation should consider not only the *E*_m_ of the electron donor but also the *E*_m_ of the electron acceptor.

However, even when this p*K*_a_ difference appears favorable, it reflects only the thermodynamic driving force. The kinetic accessibility of proton transfer depends critically on the shape of the potential energy surface along the H-bond. If the potential is nearly symmetric, indicating a LBHB (Schutz and Warshel 2004), proton transfer can proceed efficiently (Ishikita and Saito 2014). In contrast, if the potential is highly asymmetric, the proton remains predominantly localized at the donor (e.g. Mn_4_CaO_5_) with an energy barrier, and the transfer becomes energetically uphill and kinetically suppressed.

Therefore, explicit evaluation of the potential energy curve along the H-bond is essential for fully assessing both the thermodynamic and kinetic feasibility of proton transfer. This crucial requirement, frequently overlooked in many simplified theoretical studies, has been strongly emphasized in theoretical frameworks for protein proton transfer, notably by Schutz and Warshel (2004).

### Proton acceptor for O6

For O6 serving as a substrate water molecule, its deprotonation must proceed, which requires typically established H-bond network that directly links with it via H-bonds. The 2F-XFEL structure shows only one possible H-bond partner for O6, D1-Glu189 (Suga et al. 2017). Thus, D1-Glu189 was once proposed to serve as a possible proton acceptor for O6 (Suga et al. 2017). However, along the H-bond between O6 and D1-Glu189, the proton is predominantly localized at the O6 moiety, as anionic D1-Glu189 is electrostatically compensated for by cationic Mn1 (i.e. p*K*_a_(Glu189) becomes lower), disfavoring its protonation and making proton transfer from O6 energetically uphill (Mandal et al. 2020b). Thus, proposed mechanisms such as, where O6 binds as water and deprotonates upon or after incorporation, faces both energetic and mechanistic challenges.

## Spontaneous Proton Release: Mediated Via Interplay of Two Low-Barrier H-Bonds

Many theoretical studies have invested substantial effort into reproducing the incorporation of O6, largely motivated by the structural implications of 2F-XFEL data. However, in attempting to reproduce O6 incorporation starting from S_2_ atomic coordinates (e.g. 1F-XFEL structures), these studies often rely on ad hoc modeling strategies. Specifically, reaction mechanisms are frequently constructed in advance to achieve the expected outcome, and artificial forces or constraints are applied to guide water insertion into the O6 site. As such, the resulting mechanisms are *pre-biased*—their aim is not to evaluate whether O6 incorporation naturally occurs as part of the intrinsic water-splitting mechanism, but rather to enforce a predetermined outcome: namely, successful O6 incorporation as the ‘intended goal’.

In addition, many of these studies present only the relative energies of the protonated and deprotonated states as evidence for proton transfer, without examining the underlying shape of the potential energy surface along the proton transfer coordinates. Without evaluating whether the proton transfer occurs along a LBHB, or instead faces a substantial energy barrier, the kinetic feasibility of the proposed proton transfer remains ambiguous. The central scientific question is whether O6 incorporation arises spontaneously from the intrinsic energetics and structure. However, once theoretical studies begin with the assumption that O6 incorporation must occur, they tend to remain locked within that assumption. Such circular reasoning eventually inhibits progress in elucidating the water-splitting mechanism.

**Figure 2:**
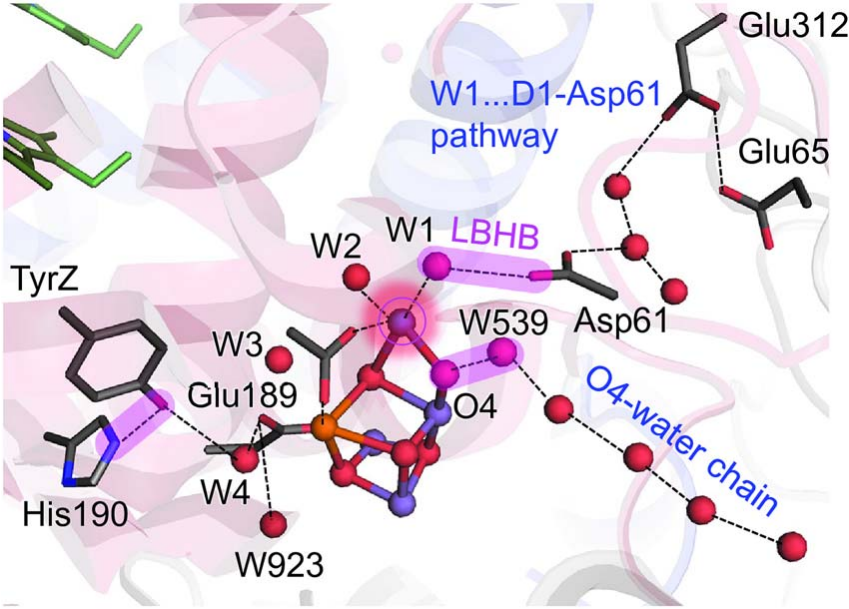
Proton transfer pathways and low-barrier H-bonds (LBHBs) near the Mn_4_CaO_5_ cluster. Three bars highlight LBHBs, which facilitate efficient proton transfer via preorganized networks. Dotted lines represent H-bonds.

Among theoretical studies, only a few have examined the S_2_ to S_3_ transition by directly increasing the oxidation state of the Mn_4_CaO_5_ cluster in S_2_, while keeping the original S_2_ atomic coordinates fully intact, without artificially guiding water insertion into the O6 site (Saito et al. 2023). One such QM/MM study revealed that efficient proton-coupled electron transfer in the S_2_ to S_3_ transition arises from the concerted action of two pre-existing LBHBs, forming an extended H-bond network spanning the TyrZ. . .Mn_4_CaO_5_. . .D1-Asp61 region (Fig. 2). Notably, throughout the QM/MM calculations, no spontaneous displacement or migration of water molecules such as W2, W3, or W4 into the O6 position was observed, in contrast to frequent assumptions in O6 incorporation models.

The first LBHB forms between TyrZ and D1-His190 (Saito et al. 2011, Kawashima et al. 2018a), while the second exists between the Mn4-bound water ligand W1 and D1-Asp61 (Kawashima et al. 2018b, Saito et al. 2020, Saito et al. 2023). This dual LBHB configuration preorganizes the system to facilitate efficient proton translocation, tightly coupling TyrZ oxidation to Mn_4_CaO_5_ deprotonation. Importantly, this mechanism proceeds without requiring substantial protein rearrangements or dynamic formation of new H-bond network, in contrast to alternative proposals that invoke proton transfer from W2, W3, W4, or O6 following conformational activation.

Upon TyrZ oxidation to TyrZ-O^•^, the LBHB with D1-His190 is disrupted. Simultaneously, nearby water molecules, W4 and an adjacent water molecule (W923) undergo only H-bond rearrangement without accompanying heavy atom displacements, ceasing to donate H-bonds to TyrZ and instead forming new H-bonds with D1-Glu189 at the Ca^2+^ site of the Mn_4_CaO_5_ cluster. This localized H-bond rearrangement lowers the p*K*_a_ of W1, initiating proton transfer from W1 to D1-Asp61 via the pre-existing W1. . .Asp61 LBHB.

This proton transfer proceeds energetically downhill and requires no significant protein dynamics, consistent with the small kinetic isotope effect [KIE ≈ 1.2 (Sakamoto et al. 2017)] observed experimentally for the initial proton release step. Following this step, protonated D1-Asp61 participates in a second H-bond network that extends toward the bulk solvent via D1-Glu65 and D2-Glu312, forming a Grotthuss-like proton wire (Fig. 2). This second phase, involving additional H-bond rearrangements, exhibits a larger KIE [~1.9 (Sakamoto et al. 2017)], reflecting its dynamically activated nature.

The overall proton transfer pathway [TyrZ. . .D1-His190]–[Mn_4_CaO_5_]–[W1. . .D1-Asp61] thus provides a long-range coupling mechanism (spanning ~ 11.4 Å) in which the Mn_4_CaO_5_ cluster functions not only as an electron donor but also as an active component in the proton transfer relay (Saito et al. 2023). This mechanism resolves several previously puzzling observations: (i) how TyrZ-O^•^ formation triggers proton release from W1, despite its spatial distance from D1-Asp61; (ii) why the initial proton transfer proceeds with minimal KIE (Shimizu et al. 2018), reflecting a pre-existing, well-organized LBHB network; (iii) why mutations at D1-Glu189 (Debus et al. 2000, Kim and Debus 2020) or substitution/removal of Ca^2+^ (Latimer et al. 1998, Kimura et al. 2002, Lohmiller et al. 2012), which coordinates W4, impair the S_2_ to S_3_ transition.

Taken together, this mechanism, centered on the interplay of two LBHBs, provides a chemically plausible, kinetically consistent, and structurally validated model for proton release during the S_2_ to S_3_ transition. It stands in sharp contrast to W2, W3, W4-, or O6-centered deprotonation models, which often invoke seemingly elegant yet, in some cases speculative, rearrangements (e.g. Bovi et al. 2013, Siegbahn 2013, Askerka et al. 2015, Shoji et al. 2015, Retegan et al. 2016, Ugur et al. 2016, Allgöwer et al. 2022, Saito et al. 2024, Tamura et al. 2025) and overlook the essential prerequisite for efficient proton transfer: a pre-formed H-bond network (Stuchebrukhov 2009). Indeed, as revealed by the electron density maps of high-resolution PSII structures (Umena et al. 2011, Suga et al. 2015), only the O4-water chain and the W1–D1-Asp61 pathway are well-structured H-bond networks capable of supporting proton transfer within the protein environment (Fig. 2). All other pathways, including those involving O6, remain speculative at the level of electron density. Further detailed structural analysis, based on electron density will be expected.

## Challenges and Future Perspectives: Revisiting the ‘O5 deprotonation trap’ in the S_0_ to S_1_ Transition

Proposed models that invoke O6 incorporation during the S_2_ to S_3_ transition inevitably treat O6 as a substrate water molecule. However, this introduces a conceptual symmetry in which O5 is likewise regarded as a substrate. These models, therefore, face a fundamental roadblock: they must account for proton release from O5 during the S_0_ to S_1_ transition, a scenario that remains extremely difficult to rationalize (Saito et al. 2015, Shimizu et al. 2018). Spectroscopic studies by Shimizu et al. indicate that the S_0_ to S_1_ transition involves a fast proton transfer process with a small KIE (≈1.2) (Shimizu et al. 2018). The transition proceeds via a single kinetic phase (~45 μs), with the rate-limiting step being electron transfer. These observations suggest that proton release occurs efficiently and without large-scale structural rearrangements.

This fast kinetics is fully consistent with QM/MM studies by Saito et al., which demonstrated that proton release in the S_0_ to S_1_ transition occurs via a pre-existing, LBHB network along the O4-water chain (Fig. 2), allowing downhill proton transfer without significant protein conformational changes (Saito et al. 2015, Takaoka et al. 2016).

In contrast, any mechanism that requires deprotonation of O5 must invoke speculative rearrangements or artificial insertion of water molecules, since O5 lacks H-bond partners in high-resolution PSII structures (Kawakami et al. 2011, Umena et al. 2011, Shen 2015). Such an H-bond configuration corresponds to a high activation barrier for proton transfer, inconsistent with the observed fast kinetics and small KIE.

A key example is Siegbahn’s widely cited model (Siegbahn 2013), which has often been referenced as theoretical support for assigning substrate status to O5. In this model, a water molecule was artificially introduced near O5 to enable deprotonation. However, this placement was achieved by twisting the D1-His332 ligand and removing the conserved hydrophobic residue D1-Val185, resulting in highly distorted geometries that deviate significantly from high-resolution PSII structures (Saito et al. 2015, Ishikita 2019). Models requiring such unrealistic structural manipulations cannot be considered physically meaningful.

This specific protein environment surrounding the Mn_4_CaO_5_ cluster effectively prevents protonation and deprotonation event of O5. A similar conclusion has been obtained in theoretical studies by Pal. et al., who could not establish H-bond formation with O5 in the presence of the PSII protein environment. Only when they removed the protein environment, mimicking Siegbahn’s manipulations, did H-bond formation with a bulk water molecule become possible (Pal et al. 2013). Thus, O5 is isolated from all H-bond networks identified in current PSII structures, including the H-bond network of the O4-water chain, as demonstrated in Saito et al. (2015).

Notably, spectroscopic studies by Shimizu et al. have demonstrated that the S_0_ to S_1_ transition is the fastest among all S-state transitions and can be explained by deprotonation of O4 rather than O5 (Shimizu et al. 2018): this interpretation appears to be reasonable, particularly because it is grounded in logic of chemistry rather than influenced by the structurally eye-catching but chemically unfavorable scenario of O5 deprotonation.

From this fundamental structural and chemical perspective, invoking O6 as a substrate water molecule implicitly requires assigning substrate status to O5 as well, thereby confronting the energetic barrier of O5 deprotonation in the S_0_ to S_1_ transition. No matter how elegantly O6 incorporation is formulated for the S_2_ to S_3_ or S_3_ to S_0_ transitions, this fundamental problem in the S_0_ to S_1_ transition remains unavoidable.

Accordingly, even if the question of O6 incorporation remains open, elucidation of the S_0_ to S_1_ mechanism should independently provide critical insight into substrate identity, serving as an essential anchor point for evaluating any proposed water-splitting mechanism. If the proton is not released from O5 in the S_0_ to S_1_ transition, as current evidence strongly suggests (Saito et al. 2015, Shimizu et al. 2018), then O5 is unlikely to serve as a substrate. And if O5 is not a substrate, then models that invoke both O5 and O6 as the substrate pair for O–O bond formation must be fundamentally reconsidered.

Unfortunately, to our knowledge, even the most detailed water-splitting mechanisms invoking O5 or O6 as substrate water molecules tend to avoid directly addressing this specific issue, as if it were ‘untouchable’. Going forward, it is essential that not only the researchers directly involved in such proposals but also reviewers and broader readers critically evaluate this issue: any proposal assuming O5 and O6 as the substrate pair remains incomplete if it fails to account for the deprotonation of O5 during the S_0_ to S_1_ transition.

This ‘*untouchable*’ situation begins to resemble Hans Christian Andersen’s *The Emperor’s New Clothes*: as long as the community refrains from directly questioning the deprotonation capability of this buried site O5 (and implicitly O6), the conceptual elegance of the O5–O6 model remains unchallenged. However, rigorous validation will ultimately be required to resolve this issue.

At present, only two well-established proton-conducting H-bond networks have been identified in the high-resolution PSII structures (Umena et al. 2011, Suga et al. 2015), each directly linking distinct oxygen sites at the Mn_4_CaO_5_ cluster (Fig. 2): (i) the O4-water chain extending from O4, located between Mn3 and Mn4 (Saito et al. 2015, Takaoka et al. 2016); and (ii) the W1. . .D1-Asp61. . .D1/Glu65/D2-Glu312 network extending from W1 at Mn4 (Kawashima et al. 2018b, Kuroda et al. 2021, Saito et al. 2023). From a protein design standpoint, especially given the limited spatial capacity for water binding at the Mn_4_CaO_5_ moiety, it seems most structurally and mechanistically plausible to utilize these two pre-existing conduits for the stepwise release of four protons over the entire Kok cycle, rather than invoking speculative or transiently formed H-bond networks, as suggested (Kawashima et al. 2018b).

Strikingly, not only these two H-bond networks, the O4-water chain and the W1. . .D1-Asp61. . .D1/Glu65/D2-Glu312 network, but a major electron transfer route exhibiting strong electronic coupling, Mn4. . .D1-Asp170. . .TyrZ (Tamura et al. 2023, Tamura et al. 2025), converge at the W1–Mn4–O4 site of the Mn_4_CaO_5_ cluster. In addition, Mn4 is the only ‘dangling’ Mn site, lacking bridging oxygen atoms, which structurally exposes this site and increases its accessibility to bulk water through the Asp61. . .D1-Glu65/D2-Glu312 network, as demonstrated in molecular dynamics simulations (Sakashita et al. 2017). Furthermore, an artificial Mn_4_CaO_4_ cluster synthesized by Zhang et al. remains stable even in the absence of the O4 site, the only μ-oxo oxygen linked not by three, but by only two metals (Mn3 and Mn4), suggesting that this oxygen, like W1 at Mn4, is flexible or exchangeable and not essential for maintaining the Mn_4_CaO_4_ core (Zhang et al. 2015).

These converging features, two proton pathways, one electron transfer route, one water intake pathway, and ligand exchangeability, all focused at the W1–Mn4–O4 site potentially highlight it as a critical hot spot for the removal of four protons and four electrons from two adjacent water molecules during oxygen evolution (e.g. Kawashima et al. 2018b).

## Data Availability

No new datasets were generated or analyzed in this study.
